# The Impact of Diabetes and Hypertension on Psychological Well-being

**DOI:** 10.1192/j.eurpsy.2025.1253

**Published:** 2025-08-26

**Authors:** N. Sghaier, I. Zariat, S. Chelly, M. Mahjoub, M. Njah, H. Ben Garouia

**Affiliations:** 1 Psychiatry Departement, Ibn Jazzar University Hospital, Kairouan; 2 Departement of hospital Hygiene, Farhat Hached University hospital, Sousse, Tunisia

## Abstract

**Introduction:**

Diabetes and hypertension are chronic conditions that significantly impact mental health. Managing these diseases involves constant monitoring, lifestyle changes, and dealing with potential complications, which can lead to increased sleep disorders, anxiety, and depression.

**Objectives:**

the objectives of our study were to determine the frequency of anxiety, depression and sleep disorders in patients with hypertension and diabetes and to investigate factors associated with their occurrence.

**Methods:**

This is a descriptive cross-sectional study conducted over a three-month period from October 1 to December 31, 2022, involving 720 diabetic and/or hypertensive patients followed at the primary health centers (CSB) in Kalaa Kbira Sousse. The assessment of sleep disorders was performed using the Pittsburgh Sleep Quality Index. Anxiety and depression were assessed using the Hospital Anxiety and Depression Scale.

**Results:**

Among the 720 patients included, the majority were women (70.6%), with a gender ratio M/F of 0.41. The average age of the participants was 63.45 ± 13.16 years. The chronic illness was hypertension in half of the cases (49.3%), diabetes in a quarter of the cases (26.3%), and both conditions together in a quarter of the cases (24.4%). Anxiety was present in nearly half of the participants (47.1%), depression affected 40.2%, while anxiety-depressive disorder concerned half of the cases (46.5%).The prevalence of sleep disorders was 54.3% according to the Pittsburgh Sleep Quality Index (PSQI) with a median score of 6 [IQR: 3-9]. The subjective quality of sleep was rated as poor in 41% of cases.The prevalence of sleep disorders was 46% among diabetics, 54.6% among hypertensive patients, and 62.5% among those with both conditions simultaneously.In multivariate analysis, factors associated with poor sleep quality according to the PSQI included age ≥ 65 years (p=0.026), comorbidities (p=0.012), anxiety-depressive disorder (p≤10^-3^), poorly controlled diabetes (p≤10^-3^), and insulin therapy (p=0.022).

**Conclusions:**

Understanding the mental health challenges faced by individuals with diabetes and hypertension is crucial for developing comprehensive care strategies that address both physical and psychological needs.

**Disclosure of Interest:**

None Declared

